# SETD2 non genomic loss of function in advanced systemic mastocytosis is mediated by an Aurora kinase A/MDM2 axis and can be therapeutically targeted

**DOI:** 10.1186/s40364-023-00468-7

**Published:** 2023-03-10

**Authors:** Manuela Mancini, Cecilia Monaldi, Sara De Santis, Cristina Papayannidis, Michela Rondoni, Chiara Sartor, Samantha Bruno, Livio Pagano, Marianna Criscuolo, Roberta Zanotti, Massimiliano Bonifacio, Patrizia Tosi, Michel Arock, Peter Valent, Michele Cavo, Simona Soverini

**Affiliations:** 1 IRCCS Azienda Ospedaliero-Universitaria Di Bologna, Istituto Di Ematologia “Seràgnoli”, Bologna, Italy; 2 Dipartimento Di Medicina Specialistica, Diagnostica E Sperimentale, Università Di Bologna, Bologna, Italy; 3 AUSL Romagna, Hematology Unit, Ravenna, Italy; 4 Divisione Di Ematologia Geriatrica Ed Emopatie Rare, Fondazione Policlinico Universitario Agostino Gemelli – IRCCS – Università Cattolica del Sacro Cuore, Roma, Italy; 5 Dipartimento Di Diagnostica Per Immagini, Fondazione Policlinico Universitario Agostino Gemelli IRCCS, Radioterapia Oncologica Ed Ematologia, Roma, Italy; 6 Section of Hematology, Multidisciplinary Outpatients Clinics for Mastocytosis, Department of Medicine, University Hospital of Verona, Verona, Italy; 7 Hematology Unit, Infermi Hospital, Rimini, Italy; 8 Department of Hematological Biology, Pitié-Salpêtrière Hospital, Pierre Et Marie Curie University (UPMC), Paris, France; 9 Department of Internal Medicine I, Division of Hematology and Hemostaseology, Medical University of Vienna, Vienna, Austria; 10 Ludwig Boltzmann Institute of Hematology and Oncology, Medical University of Vienna, Vienna, Austria

**Keywords:** SETD2, MDM2, Aurora kinase A, Polo-like kinase 1, Proteasome inhibitors

## Abstract

**Background:**

The *SETD2* tumor suppressor gene encodes a histone methyltransferase that safeguards transcription fidelity and genomic integrity via trimethylation of histone H3 lysine 36 (H3K36Me3). *SETD2* loss of function has been observed in solid and hematologic malignancies. We have recently reported that most patients with advanced systemic mastocytosis (AdvSM) and some with indolent or smoldering SM display H3K36Me3 deficiency as a result of a reversible loss of SETD2 due to reduced protein stability.

**Methods:**

Experiments were conducted in SETD2-proficient (ROSA^KIT D816V^) and -deficient (HMC-1.2) cell lines and in primary cells from patients with various SM subtypes. A short interfering RNA approach was used to silence *SETD2* (in ROSA^KIT D816V^ cells), *MDM2* and *AURKA* (in HMC-1.2 cells). Protein expression and post-translational modifications were assessed by WB and immunoblotting. Protein interactions were tested by using co-immunoprecipitation. Apoptotic cell death was evaluated by flow cytometry after annexin V and propidium iodide staining, respectively. Drug cytotoxicity in in vitro experiments was evaluated by clonogenic assays.

**Results:**

Here, we show that the proteasome inhibitors suppress cell growth and induce apoptosis in neoplastic mast cells by promoting SETD2/H3K36Me3 re-expression. Moreover, we found that Aurora kinase A and MDM2 are implicated in SETD2 loss of function in AdvSM. In line with this observation, direct or indirect targeting of Aurora kinase A with alisertib or volasertib induced reduction of clonogenic potential and apoptosis in human mast cell lines and primary neoplastic cells from patients with AdvSM. Efficacy of Aurora A or proteasome inhibitors was comparable to that of the KIT inhibitor avapritinib. Moreover, combination of alisertib (Aurora A inhibitor) or bortezomib (proteasome inhibitor) with avapritinib allowed to use lower doses of each drug to achieve comparable cytotoxic effects.

**Conclusions:**

Our mechanistic insights into SETD2 non-genomic loss of function in AdvSM highlight the potential value of novel therapeutic targets and agents for the treatment of patients who fail or do not tolerate midostaurin or avapritinib.

**Supplementary Information:**

The online version contains supplementary material available at 10.1186/s40364-023-00468-7.

## Introduction

Systemic mastocytosis is a rare hematological neoplasm characterized by clonal expansion and accumulation of morphologically and immunophenotypically abnormal mast cells in extracutaneous tissues and organs including the bone marrow, spleen, liver and gastrointestinal tract [1–4]. From a clinical point of view, SM is a highly heterogeneous disease with clinical presentations ranging from indolent variants with near-normal life expectancy to more aggressive entities, the so-called advanced SM [2–4]. AdvSM includes SM with an associated hematologic neoplasm (SM-AHN), aggressive SM (ASM) and MC leukemia (MCL). While patients with indolent SM (ISM) and smoldering SM (SSM) generally do not require cytoreductive treatment since only a few of them will eventually progress to ASM, patients with AdvSM often progress and may have rapidly deteriorating courses with poor outcome [2–4]. Since constitutive activation of the KIT tyrosine kinase receptor as a consequence of gain-of-function mutations in the *KIT*gene (most frequently D816V) is considered a disease-driving event in the pathogenesis of SM [5–9], the multikinase inhibitor midostaurin, that targets both wild-type and mutant KIT, has been tested in clinical trials and recently approved for use in patients with AdvSM [10, 11]. However, a significant proportion of patients do not respond to or relapse on midostaurin [6, 10]. Tolerability issues may also lead to discontinuation of treatment. More recently, the KIT D816V-targeting drug avapritinib has been developed and has received approval by the European Medicines Agency for use in AdvSM patients after at least one systemic therapy [12, 13]. However, not all patients with AdvSM may respond to or tolerate this kinase blocker.

Efforts aimed to identify novel molecular players cooperating with *KIT*gain-of-function mutations in the pathogenesis of SM have recently led us to the discovery of a SETD2 protein deficiency in the MCL-like human MC lines HMC-1 and patients with SM. SETD2 deficiency was found to correlate roughly with the WHO category and disease aggressiveness in SM [14]. SETD2 is encoded by a tumor suppressor whose loss of function (via biallelic inactivating mutations, or monoallelic deletions or copy neutral loss of heterozygosity and mutation of the remaining allele) has been observed in a variety of solid tumors and, more recently, in hematologic malignancies of both myeloid and lymphoid origin [15]. SETD2 is a methyltransferase that catalyzes the trimethylation of histone H3 at lysine 36 [16], a key epigenetic modification that can be used as a surrogate marker of SETD2 loss of function. We have recently shown that SETD2 loss of function in SM mainly occurs at the post-translational rather than at the genomic level, via hyperubiquitination and proteasomal degradation – being thus potentially reversible. Indeed, proteasome inhibition by bortezomib rescued SETD2 expression and restored H3K36Me3 in neoplastic MCs. In this study, we aimed to dissect the upstream mechanisms responsible for SETD2 loss of function in SM to define novel therapeutic targets and agents (possibly via repurposing of drugs already approved or in advanced clinical development) for the treatment of AdvSM patients who fail or do not tolerate midostaurin and/or avapritinib.

## Methods

### Patient samples and cell lines

Experiments were conducted in SETD2-proficient (ROSA^KIT D816V^) and -deficient (HMC-1) cell lines [17–19] and in primary cells from patients with various SM subtypes. Cell lines and patient characteristics are detailed in the Supplementary methods. Sample collection was approved by the Institutional Review Boards of the S. Orsola-Malpighi Hospital (protocol 112/2014/U/Tess) and of the other institutions participating in this study.

### RNA interference

A short interfering RNA approach was used to silence *SETD2* (in ROSA^KIT D816V^ cells), *MDM2* and *AURKA* (in HMC-1 cells) as described in the Supplementary methods.

### Co-immunoprecipitation/immunoblotting and Western blot analyses

HMC-1 and ROSA^KIT D816V^ cells maintained in control medium or after 24 h-incubation with 10 nM bortezomib, 5 nM carfilzomib, 50 nM ixazomib and 5 µM SP141 were lysed to extract total cellular proteins. WB, co-immunoprecipitation and immunoblotting were performed as described in the Supplementary methods. The following primary antibodies were used for Co-IP/immunoblotting and WB: anti-SETD2 (goat polyclonal antibody, cat. PAB7385, Abnova), anti-H3K36Me3 (rabbit monoclonal antibody, clone D5A7 cat. 4909) anti-p53 (rabbit monoclonal antibody, clone 7F5 cat.2527), anti-Mdm2 (rabbit monoclonal antibody, cloneD1V2Z, cat. 86,934), anti-ubiquitin (rabbit monoclonal antibody, clone E6K4Y, cat. 20,326), anti-Aurora kinase A (rabbit monoclonal antibody, clone D3V7T, cod. 91,590, anti-phospho-Aurora kinase A(T288) (rabbit monoclonal antibody, clone C39D8, cod. 3079), anti-Plk1 (rabbit monoclonal antibody, clone208G4, cod. 4513), anti phospho-Plk1(T210) (rabbit polyclonal antibody, cat. 5472), anti-phospho-Ser/Thr (rabbit polyclonal antibody, cat. 9631)(all from Cell Signaling Technology). Beta-actin (mouse monoclonal antibody, clone AC-15, cod. SC-69879 Santa Cruz biotechnology) or beta-tubulin (rabbit monoclonal antibody, clone 3F7, cod. 2128 Cell Signaling Technology) were used as loading control. Immunoreactive proteins were visualized by probing with horseradish peroxidase-conjugated secondary antibodies and then by enhanced chemiluminescence (ECL, Thermo Fisher Scientific). Signal intensities in single blots obtained from three individual experiments were quantified with the ImageJ software, which attributes a numerical value to signals of chemiluminescent substrates, thereby allowing a comparative analysis of protein levels across different samples.

### Clonogenic assays

Clonogenic assays were used to evaluate anti-neoplastic drug effects in HMC-1.1 and 1.2 cells, in siSETD2 ROSA^KIT D816V^ cells and in primary cells from AdvSM patients, according to procedures described in Supplementary methods. Drug specificity was evaluated using healthy donor cells as control.

### Apoptosis

After 24 h exposure to bortezomib (10 nM), carfilzomib (5 nM), ixazomib (50 nM), alisertib, volasertib (500 nM) and avapritinib (250 nM) alone or in combination with bortezomib or alisertib, apoptotic cell death was assessed in HMC-1 and ROSA^KIT D816V^ cells by using the Annexin-V-FLUOS Staining Kit by Roche Applied Science, according manufacturer instructions and measuring the uptake of fluorescinated Annexin V and propidium iodide (both from Roche). A FACSCanto II flow cytometer (Beckton Dickinson) set at 488 nm excitation and 530 nm wavelength bandpass filter for fluorescein detection or 580 nm for PI detection and a dedicated software (DIVA, Beckton Dickinson) were used for analysis.

### Statistical analysis

Data are presented as mean values ± standard deviation (SD) and were analyzed for statistical significance by Student t-test (GraphPad Prism).

## Results

### The efficacy of proteasome inhibitors in reducing colony growth and inducing apoptosis is SETD2/H3K36Me3-dependent

**Fig. 1:**
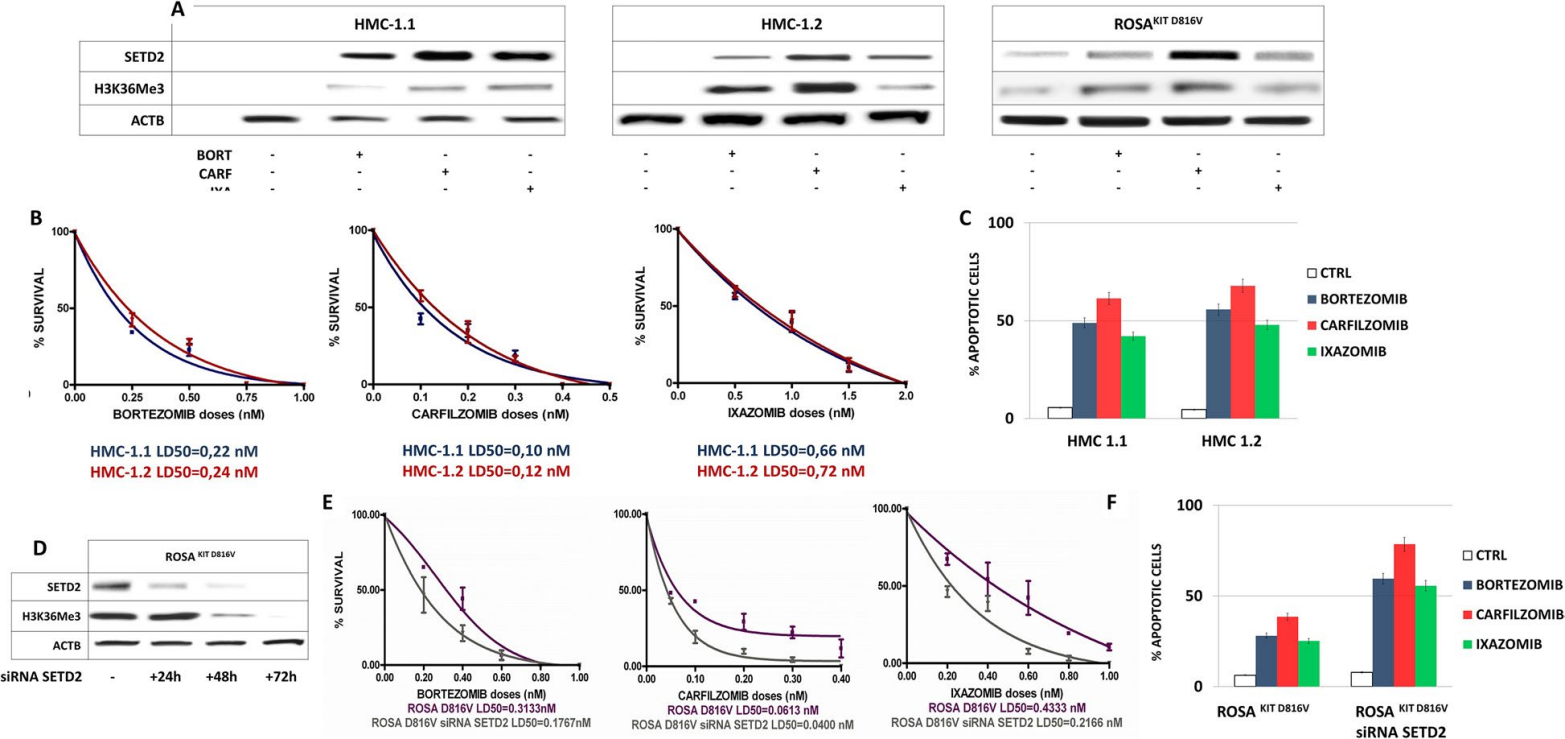
The cytotoxic effects of proteasomal inhibition are SETD2/H3K36Me3-dependent. **A** Effect of proteasome inhibition by bortezomib (bort), carfilzomib (carf) and ixazomib (ixa) on SETD2 and H3K36Me3 levels in HMC-1.1, HMC-1.2 and ROSA^KIT D816V^. **B** Reduction of clonogenic growth of HMC-1.1 and -1.2 cells in the presence of increasing doses of bortezomib, carfilzomib and ixazomib. All the clonogenic survival rates are expressed as mean ± standard deviation of counts from three independent experiments. **C** Induction of apoptosis in HMC-1.1 and -1.2 cells after 24 h-incubation with 10 nM bortezomib, 5 nM carfilzomib and 50 nM ixazomib. Each column represents the mean of three independent experiments and the bars represent the standard error. **D** Reduction of SETD2 expression and H3K36Me3 after 24, 48 and 72 h of SETD2 depletion by siRNA. **E** Reduction of clonogenic growth of control and SETD2 RNAi-depleted ROSA^KIT D816V^ cells in the presence of increasing doses of bortezomib, carfilzomib and ixazomib. **F** Induction of apoptosis in control and SETD2 RNAi-depleted ROSA^KIT D816V^ cells after 24 h-incubation with 10 nM bortezomib, 5 nM carfilzomib and 50 nM ixazomib

We have previously reported that bortezomib is strikingly effective in reducing colony growth and inducing apoptosis both in SETD2/H3K36Me3-deficient HMC-1.1 and -1.2 cells and in neoplastic MCs from SETD2/H3K36Me3-deficient MCL patients [14]. To explore further the potential of proteasome inhibition as a therapeutic option for AdvSM, the efficacy of two second-generation inhibitors already approved for clinical use (carfilzomib and orally available ixazomib) was investigated. Like bortezomib [14], carfilzomib and ixazomib restored SETD2 expression and activity (as shown by rescue of H3K36Me3) in HMC-1.1 and -1.2 cells after 24 h of incubation at 5 nM and 50 nM, respectively (Fig. 1A). Clonogenic assays in HMC-1 cells showed that bortezomib, carfilzomib and ixazomib were similarly active in inducing a dose-dependent reduction in colony growth, with LD_50_ in the subnanomolar range (HMC-1.1: 0.22 nM, 0.10 nM and 0.66 nM; HMC-1.2: 0.24 nM, 0.12 nM and 0.72 nM, respectively) (Fig. 1B). Moreover, Annexin V-PI double staining demonstrated that 24 h-incubation with 10 nM of bortezomib, 5 nM of carfilzomib or 50 nM of ixazomib induced apoptosis in a relevant proportion of cells (45–70%), with carfilzomib being the most active agent (Fig. 1C).

To assess whether the activity of proteasome inhibitors was dependent on SETD2 expression, we next performed clonogenic and apoptosis assays in ROSA^KIT D816V^ cells before and after SETD2 RNAi depletion. WB confirmed that SETD2 and H3K36Me3 levels progressively decreased in ROSA^KIT D816V^ cells after 24 and 48 h and disappeared after 72 h of silencing (Fig. 1D). The extent of reduction in clonogenic potential in the presence of bortezomib, carfilzomib or ixazomib was greater in SETD2 RNAi-depleted cells as compared to control cells (Fig. 1E). Similarly, the percentage of apoptotic cells after proteasome inhibitor treatment was significantly greater in SETD2 RNAi-depleted cells (Fig. 1F).

**Fig. 2:**
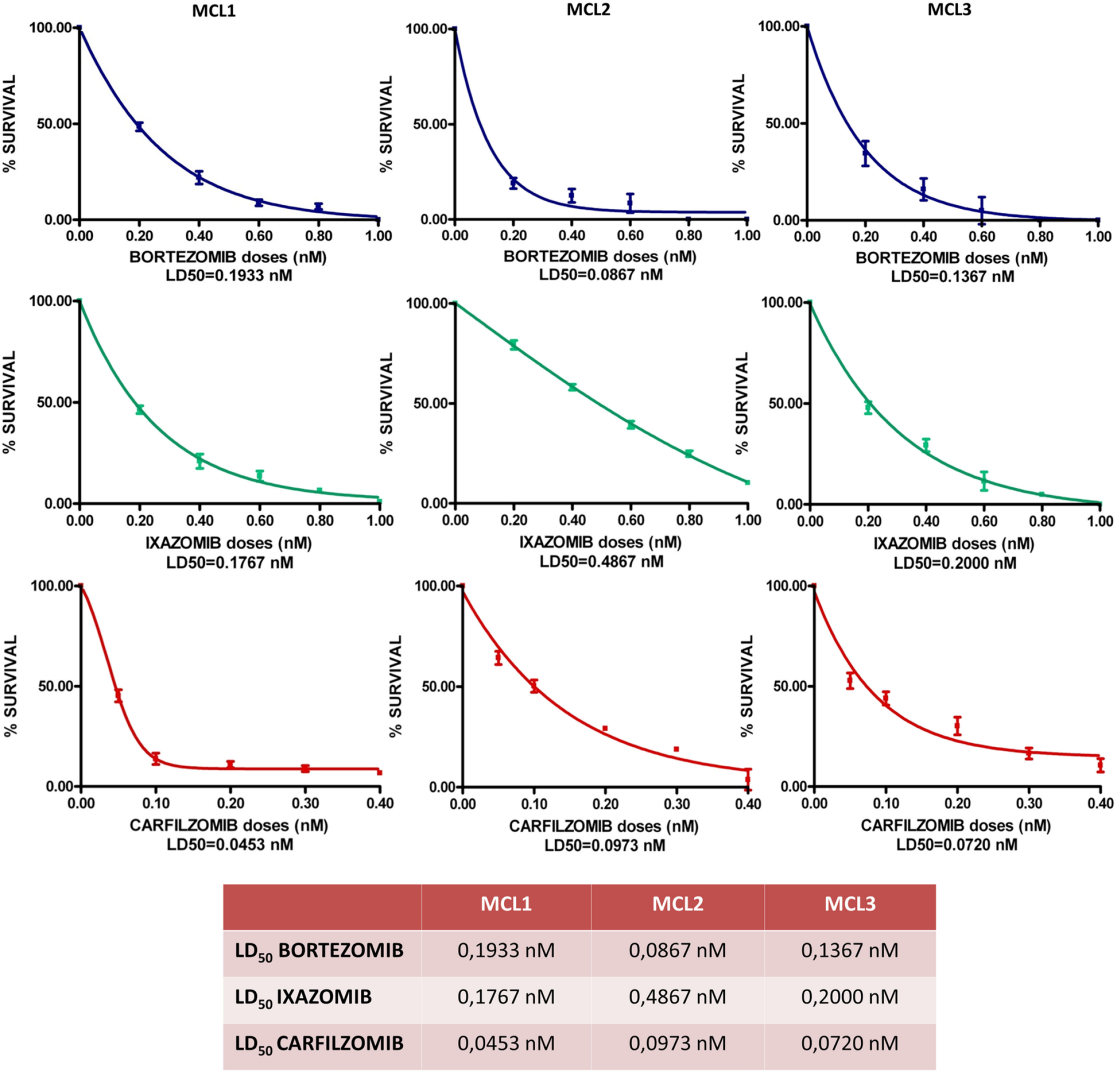
Reduction of clonogenic potential of neoplastic MCs from 3 patients with MCL in the presence of increasing doses of bortezomib, ixazomib and carfilzomib. All the clonogenic survival rates are expressed as mean ± standard deviation of counts from two independent experiments

To confirm the results obtained in our in vitro experiments, we next assessed the differences in response to bortezomib, carfilzomib and ixazomib in 3 SETD2/H3K36Me3-deficient MCL patients. To this purpose, we evaluated drug impact on clonogenic activity. Dose–response curves and drug LD_50_ are shown in Fig. 2. Although all the tested drugs demonstrated a clonogenic inhibitory activity at subnanomolar doses, carfilzomib showed the lowest LD_50_.

### SETD2 interacts with p53 and is ubiquitinated by MDM2, whose inhibition rescues SETD2 expression and function

**Fig. 3:**
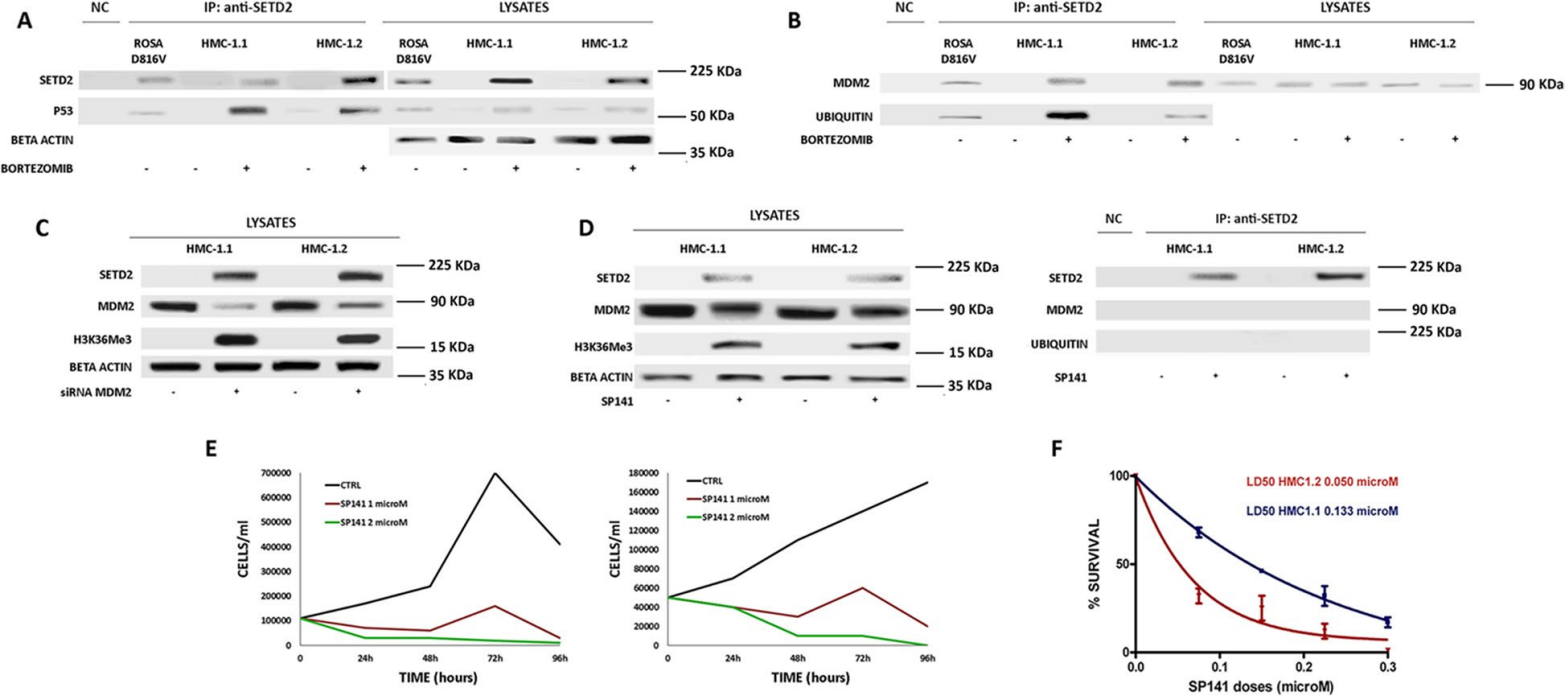
Effects of MDM2 inhibition on SETD2 expression and function. Co-immunoprecipitation assays showed that (**A**) p53 interacts with SETD2 in the SETD2-proficient ROSA^KIT D816V^ cell line and, after bortezomib treatment, in SETD2-deficient HMC-1 cells, and that (**B**) ubiquitinated SETD2 binds MDM2 in the same context. **C** and **D** Silencing or inhibiting MDM2 by siRNA (for 72 h) or by SP141 (5 µM for 24 h), respectively, rescued SETD2/H3K36Me3 expression in HMC-1 cells. A negative control (NC) obtained by the immunoprecipitation of protein lysates using a resin conjugated with an anti-IgG1 antibody was loaded for each immunoprecipitation experiment. **E** SP-141 administration in HMC-1 cells (red: 1 µM; green: µM) maintained in liquid medium and counted every 24 h up to 96 h of treatment showed cytostatic effects. **F** Clonogenic assays performed in semisolid medium using scalar doses of the drug (0.075–0.3 µM) supported the cytostatic effects of SP141 in HMC-1.1 and -1.2 cell lines

SETD2 has been reported to bind p53 in HEK293T and MCF7 cells [20] and to be necessary for its activation in clear cell renal cell carcinoma[21]. We thus investigated whether the same occurs in AdvSM. Indeed, we found that p53 co-immunoprecipitates with SETD2 in the SETD2-proficient ROSA^KIT D816V^ cell line (Fig. 3A). In addition, we observed the same results, after proteasome inhibition, in the SETD2-deficient HMC-1 MCL cell line (Fig. 3A). Having demonstrated that p53 and SETD2 physically interact in SM cell line models and knowing that p53 is ubiquitinated by MDM2, we hypothesized that MDM2 might be the ubiquitin-E3-ligase responsible for SETD2 hyper-ubiquitination, triggering its proteasome-mediated degradation in AdvSM. Indeed, we found that MDM2 co-immunoprecipitates with hyper-ubiquitinated SETD2 in ROSA^KIT D816V^ cells as well as in HMC-1.1 and -1.2 cells after proteasome inhibition (Fig. 3B).

SiRNA-mediated knock-down of MDM2 was found to rescue SETD2 expression and function in HMC-1 cells, as shown by WB (Fig. 3C). The same was observed after treatment for 24 h with 5 µM SP-141, a small molecule belonging to a new class of MDM2 inhibitors that promote MDM2 auto-ubiquitination and degradation (Fig. 3D) [22–24]. Taken together, these findings point to MDM2 as the main ubiquitin-protein ligase responsible for SETD2 hyperubiquitination in AdvSM. Given that interfering with SETD2 proteasome-mediated degradation by bortezomib, carfilzomib or ixazomib was found to be a promising therapeutic approach, and that SP-141, in contrast to many other MDM2 inhibitors, acts directly on MDM2 stability rather than on MDM2-p53 interaction, we asked whether targeting MDM2-mediated hyperubiquitination of SETD2 by SP-141 could be an additional therapeutic approach in AdvSM contexts. Treatment with the MDM2 inhibitor SP-141 rescued SETD2 expression and function in HMC-1 cells. However, SP-141 treatment of HMC-1 cells at micromolar doses induced cytostatic but not cytotoxic effects as shown by cell growth curves (Fig. 3E). Clonogenic assays supported the cytostatic effects of SP141 in HMC-1.1 and -1.2 cells (Fig. 3F), since LD_50_ were rather high (0.050 and 0.133 µM, respectively) and even at the maximum doses the clonogenic potential was not fully suppressed.

### Aurora kinase A is overexpressed and hyperactivated in neoplastic cells in AdvSM, phosphorylates SETD2 and contributes to its proteasome-mediated degradation

**Fig. 4:**
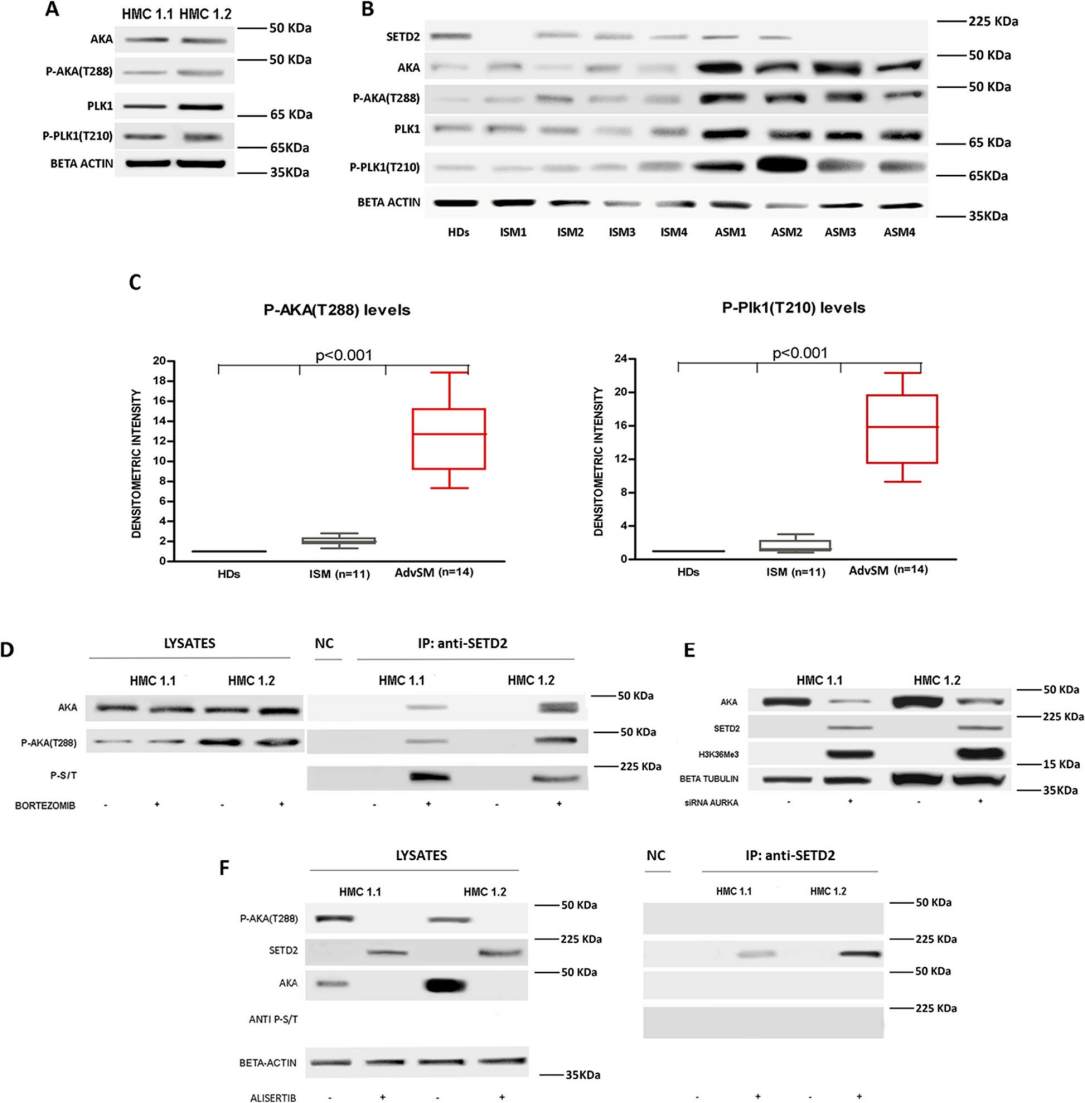
The Aurora kinase A/Plk1 axis is hyper-activated in AdvSM and affects SETD2/H3K36Me3 expression. **A** Aurora A and Plk1 expression and phosphorylation in HMC-1 cells assessed by WB. **B** Representative Western blot results for Aurora A and Plk1 protein expression and phosphorylation in SM patients (4 ISM and 4 ASM) as compared to a pool of healthy donors (HDs). **C** Box plots of phospho-Aurora A and phospho-Plk1 [P-AKA(T288) and P-Plk1(T210)] expression levels estimated by densitometric analysis of Western blots in 11 ISM patients (grey) and 14 AdvSM patients (red). Signal intensities in single blots obtained from three individual experiments were quantified in comparison to the signal intensities obtained for the pool of HDs, used as control. **D** Co-immunoprecipitation assay showing that total and phospho-Aurora A can be found bound to serine/threonine phosphorylated SETD2 after bortezomib treatment in the SETD2-deficient HMC-1 cells. **E** and **F** Silencing or inhibiting Aurora A by siRNA (for 72 h) or by alisertib (500 nM for 24 h), respectively, induced SETD2 release from Aurora A and de-phosphorylation, and resulted in the rescue of SETD2 expression in HMC-1 cells. A negative control (NC) obtained by the immunoprecipitation of protein lysates using a resin conjugated with an anti-IgG1 antibody was loaded for each immunoprecipitation experiment

In an attempt to identify additional key players implicated in SETD2 hyperubiquitination and proteasome-mediated degradation, we interrogated a series of protein–protein interaction databases. Interestingly, both BioGRID (https://thebiogrid.org/) [25] and IntAct (https://www.ebi.ac.uk/intact/) [26] returned Aurora kinase A serine/threonine kinase among SETD2 interactors. Accordingly, NetworKIN (http://www.networkin.info/)[27, 28], an integrative computational approach that links experimentally identified phosphorylation sites to protein kinases on the basis of consensus sequence motifs as well as contextual information, identified several phospho-serine and -threonine sites that are predicted to be Aurora kinase A targets with a high confidence score. Bearing in mind that phosphorylation is often the trigger for ubiquitination and consequent proteasome-mediated degradation, we set out to investigate whether Aurora kinase A may be implicated in SETD2 non genomic loss of function in AdvSM. First, we performed Western blot experiments to examine the expression and phosphorylation status of Aurora A and Polo like kinase 1 (Plk1) a serine/threonine kinase that is known to be engaged with Aurora kinase A [29] in HMC-1.1 and -1.2 cells and in primary cells from patients with ISM (*n* = 11) and ASM (*n* = 14). Both Aurora kinase A and Plk1 were found to be overexpressed and activated in HMC-1 cells (Fig. 4A) and in 14 patients with AdvSM as compared to patients with ISM, who displayed protein and phosphorylation levels superimposable to those of healthy donors. Overall, Aurora kinase A and Plk1 expression and activation inversely correlated with SETD2 expression. A representative WB experiment is shown in Fig. 4B, while results obtained in the entire cohort of patients are summarized in Fig. 4C. In HMC-1 cells, co-immunoprecipitation performed after proteasome inhibition showed that Aurora kinase A indeed binds a serine/threonine-phosphorylated form of SETD2 (Fig. 4D). SiRNA-mediated knock-down of Aurora kinase A rescued SETD2 expression and function (Fig. 4E). Similar effects were observed with pharmacological inhibition of Aurora A kinase activity using alisertib. When Aurora kinase A activity was turned off, SETD2 and Aurora kinase A binding, as well as SETD2 serine/threonine phosphorylation, were abrogated (Fig. 4F). Taken together, these findings support the hypothesis that Aurora kinase A is implicated in SETD2 non genomic loss of function in AdvSM.

### Aurora kinase A and Plk1 inhibition with alisertib and volasertib, respectively, reduce colony growth and induce apoptosis

**Fig. 5:**
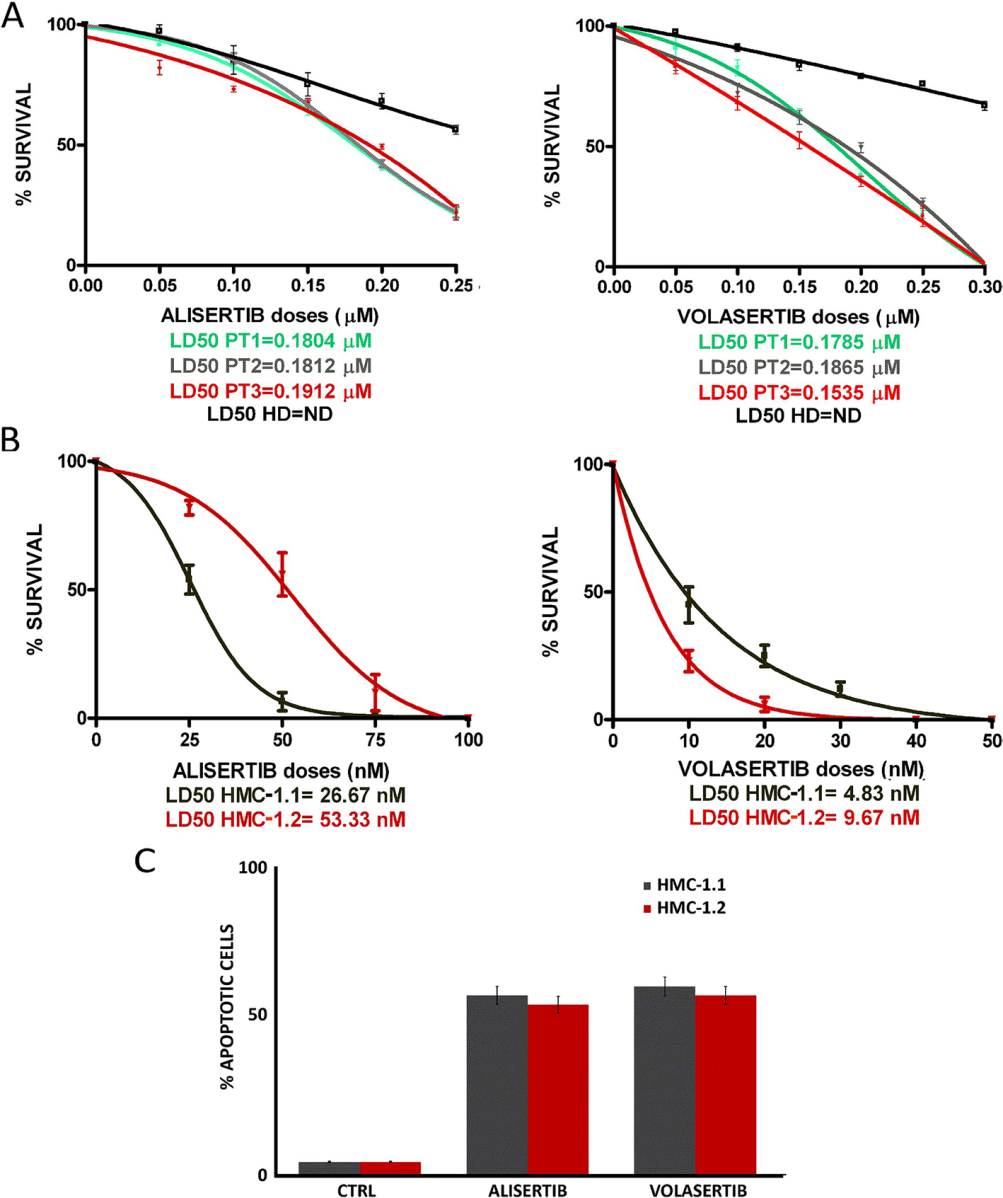
Effects of alisertib and volasertib in HMC-1 cells and in primary MCL cells. **A** Reduction of clonogenic potential of neoplastic MCs from 3 patients with MCL (green, grey and red curves) as compared to a healthy donor (HD, black curve) in the presence of increasing doses of alisertib (0.05–0.25 µM) or volasertib (0.05–0.30 µM). **B** Reduction of clonogenic growth of HMC-1.1 (grey) and 1.2 (red) cells in the presence of increasing doses of alisertib or volasertib (25–100 nM). All the clonogenic survival rates are expressed as mean ± standard deviation of counts from three independent experiments. **C** Induction of apoptosis by alisertib or volasertib (500 nM for 24 h) in HMC-1.1 (grey) and -1.2 (red), respectively. Columns represent the mean of three independent experiments and bars represent the standard error

We next investigated whether Aurora kinase A and Plk1 inhibition could be a useful therapeutic strategy for AdvSM patients. To address this point, we assessed the effects of alisertib and volasertib on the clonogenic potential of neoplastic MCs from 3 MCL patients (Fig. 5A) and of HMC-1 cells (Fig. 5B). We found that both drugs significantly reduced the proliferation of the HMC-1 cell lines and of primary neoplastic cells in all patients tested. Importantly, drug treatments only partially affected the viability of cells derived from a healthy donor, tested as control.

Flow cytometry analysis of apoptosis induction in HMC-1 cells following 24 h treatment with alisertib or volasertib (500 nM each) showed that cell lines were sensitive to both drugs reaching remarkable apoptotic levels at submicromolar doses (Fig. 5C). Collectively, these findings suggest that administration of Aurora kinase A or Plk1 inhibitors could be a promising therapeutic strategy in AdvSM patients.

### In vitro, Aurora A inhibition and proteasome inhibition efficacy compares to that of avapritinib in SETD2-deficient cells

**Fig. 6:**
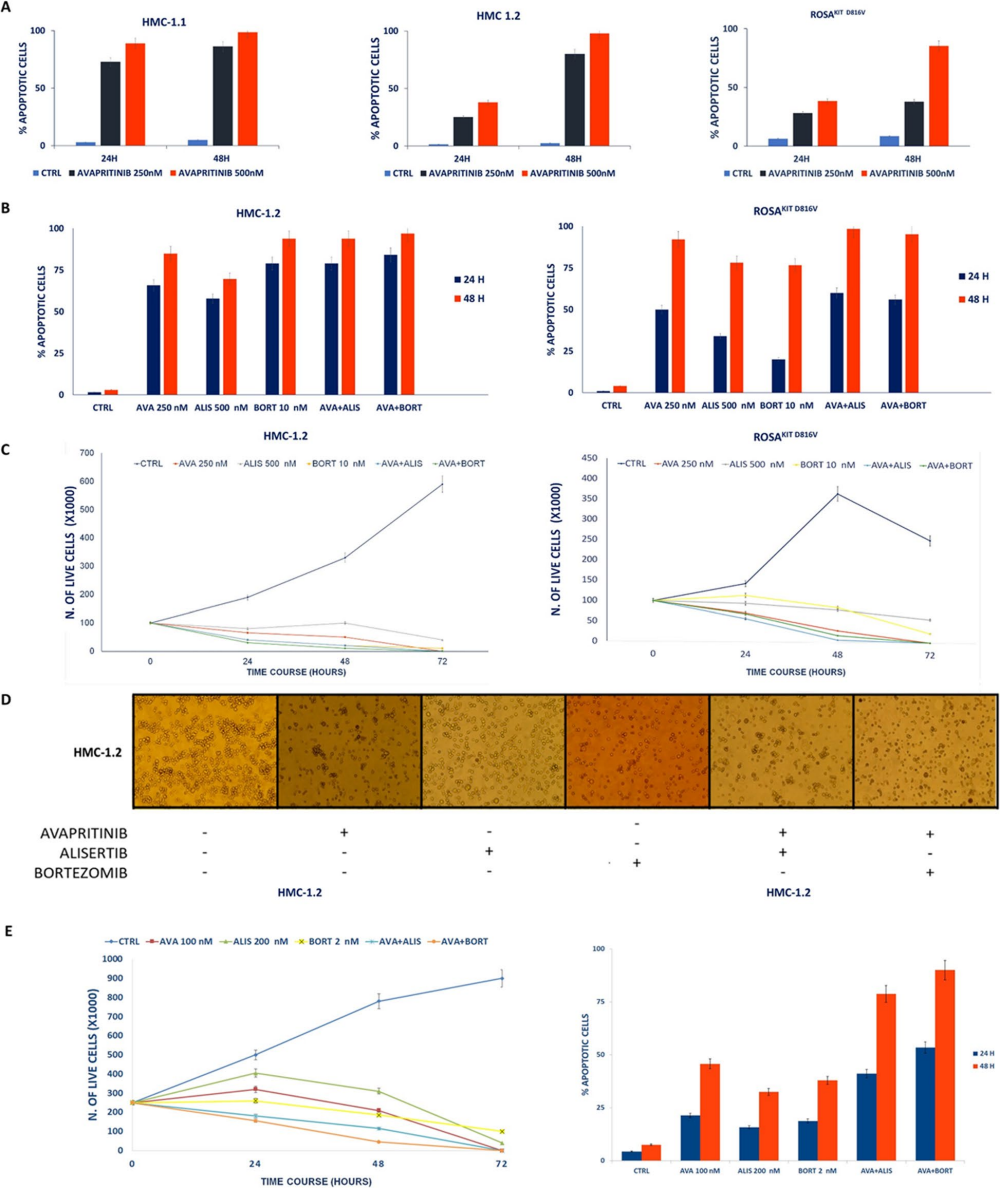
Effects of avapritinib, alisertib and bortezomib in SM cell lines. **A** Induction of apoptosis by alisertib 250 (blue) and 500 (orange) nM used as single agent after 24 and 48 h of treatment in HMC-1.1, -1.2 and in ROSA^KIT D816V^ cells. Columns represent the mean of three independent cytofluorimetric assays performed by loading cells with Annexin V (FITC) and PI and bars represent the standard error. **B** Induction of apoptosis by alisertib (500 nM) or bortezomib (10 nM) alone and in combination with avapritinib (250 nM) after 24 (lblue) and 48 (orange) hours of treatment in HMC-1.2 and ROSA^KIT D816V^ cells. Columns represent the mean of three independent cytofluorimetric assays performed by loading cells with Annexin V (FITC) and PI and bars represent the standard error. **C** Avapritinib (250 nM, orange), alisertib (500 nM, grey) and bortezomib (10 nM, yellow) administration, alone and in combination (AVA + ALIS, light blue; AVA + BORT, green) in HMC-1.2 and ROSA^KIT D816V^ cells maintained in liquid medium and counted every 24 h up to 72 h of treatment showed that the combinations did not significantly potentiate the cytotoxic effects of either drug alone. Each point represents the mean of three independent counts performed after trypan blue staining by using the burker chamber. **D** Induction of cell death by avapritinib (250 nM) and alisertib (500 nM) or bortezomib (10 nM) treatment in HMC-1.2 cells assessed by using an EVOS XL Core optical microscope equipped with a 20 × objective. **E** Avapritinib (100 nM, red), alisertib (200 nM, green) and bortezomib (2 nM, yellow) administration, alone and in combination (AVA + ALIS, light blue; AVA + BORT, orange) in HMC-1.2 cells maintained in liquid medium and counted every 24 h up to 72 h of treatment showed that the combinations, at low doses of each drug, significantly potentiate the cytotoxic effects of either drug alone. Each point represents the mean of three independent counts performed after trypan blue staining by using the burker chamber

KIT D816V is the main disease driver in SM, and avapritinib is the most potent KIT D816V inhibitor currently available in the clinic. We thus decided to compare the cytotoxic effects of Aurora kinase A and proteasome inhibition to those of avapritinib, and to investigate the efficacy of combination strategies. First, we tested the cytotoxic effects of avapritinib in HMC-1 and ROSA^KIT D816V^ cells. Flow cytometry analysis of apoptosis after 24 and 48 h of treatment with avapritinib (250 nM and 500 nM) demonstrated marked cytotoxic effects at submicromolar doses (Fig. 6A). Efficacy of avapritinib in inducing apoptosis in SETD2-deficient HMC-1.2 cells was comparable to that of alisertib and was slightly inferior to that of bortezomib (Fig. 6B-C-D). In contrast, avapritinib showed a significant advantage if compared to alisertib and bortezomib in SETD2-proficient ROSA^KIT D816V^ cells (Fig. 6B-C). The association of avapritinib and alisertib (250 + 500 nM) or avapritinib and bortezomib (250 + 10 nM) did not significantly potentiate the cytotoxic effects of either drug alone (Fig. 6B-C). Interestingly, however, using lower doses of each drug (avapritinib 100 nM, alisertib 200 nM and bortezomib 2 nM) in SETD2-deficient HMC-1.2 cells we observed a clear advantage of the combination treatments as early as 24 h after the administration (Fig. 6E).

## Discussion

H3K36Me3 has emerged as a particularly important chromatin regulator safeguarding genomic integrity and fidelity of gene expression. Thus, it is not surprising that its deregulation – mainly due to deficient SETD2 methyltransferase activity – has been implicated in diverse forms of cancer. We have recently shown that SETD2 function is reduced or lost in neoplastic MCs of some ISM and SSM patients and in virtually all those with AdvSM, as well as in MCL-related cell lines. We have also observed that in SM, SETD2 loss of function most frequently results from reduced protein stability rather than from gene mutations, deletions, loss of heterozygosity or combinations of these genetic events, as it happens in solid tumors [14]. The present study builds on and extends our previous findings in SM by providing evidence for a mechanism that underlies SETD2 non genetic loss of function and may involve Aurora kinase A and MDM2.

Since in the great majority of AdvSM patients SETD2 loss of function occurs at the post-translational level, it is reversible. Accordingly, we have shown that interfering with proteasome-mediated degradation could rescue the expression of a functional SETD2 protein [14]. In MCL cell lines and in neoplastic MCs from MCL patients, bortezomib was found to be strikingly effective, even at subnanomolar concentrations, in inducing apoptosis and inhibiting clonogenic potential [14]. The efficacy of bortezomib, alone or in combination with midostaurin, had already been reported in a study by Aichberger et al. [30] Here, we show that the second-generation proteasome inhibitors carfilzomib and ixazomib (both approved, like bortezomib, for the treatment of multiple myeloma – hence easily repurposable) are similarly effective in cell lines and primary patient cells, with carfilzomib being the most potent drug. Additionally, we provide mechanistic insights into the mode of action of proteasome inhibitors in AdvSM. In fact, we show that the efficacy of these proteasome inhibitors is SETD2-dependent, since turning off SETD2 in a SETD2-proficient SM cell line (ROSA^KIT D816V^) increased substantially the percentage of apoptotic cells after exposure to bortezomib, carfilzomib or ixazomib. This further strengthens the hypothesis that reverting SETD2 deficiency is a valuable therapeutic strategy and prompted us to search for druggable players implicated in the aberrant degradation of SETD2 in neoplastic MCs. The observation that SETD2 is capable of binding p53 in several cell line models^20,21^as well as in primary neoplastic MCs highlighted MDM2 as a potential candidate ‘culprit’ for aberrant SETD2 ubiquitination. Both RNAi knock-down and pharmacological suppression of MDM2 rescued SETD2 expression in neoplastic MCs, indicating that MDM2 is directly or indirectly implicated in SETD2 non genomic loss of function in AdvSM. Since we also observed that hyperubiquitinated SETD2 can be found bound to MDM2 in HMC-1 cells after proteasome inhibition, a direct role for MDM2 seems plausible. When we moved on to search for an upstream trigger of SETD2 aberrant ubiquitination, our attention was captured by Aurora kinase A, that has been reported to be a SETD2 interactor by a high-throughput study of protein interaction networks [31]. Aurora kinase A is a serine and threonine kinase that associates with the centrosome and the spindle microtubules and plays important roles during mitosis and cytokinesis [29]. Intriguingly, SETD2 has recently been reported to associate with spindle microtubules, too, where it methylates α-tubulin at lysine 40 during mitosis [32]. Several phospho-serine and -threonine sites of SETD2 lie within Aurora kinase A consensus sequences. We experimentally verified that: i) Aurora kinase A is overexpressed at the protein level and activated in all the SM cases showing SETD2 deficiency, but not in cases with SETD2 levels superimposable to healthy donors; ii) Aurora kinase A immunoprecipitates with serine/threonine-phosphorylated SETD2 after proteasome inhibition; iii) both siRNA-mediated silencing and pharmacological inhibition of Aurora kinase A activity rescues SETD2 and H3K36Me3; iv) inhibition of Aurora A kinase activity not only disrupts Aurora kinase A binding to SETD2 but also serine/threonine phosphorylation of SETD2. Taken together, these findings point to a role for Aurora kinase A in SETD2 non genomic loss of function in SM. Importantly, this role offers additional therapeutic opportunities. First of all, alisertib – a specific Aurora kinase A inhibitor currently being evaluated in clinical trials in various neoplastic conditions, including acute myeloid leukemia [33–35] demonstrated very good preclinical activity when we tested it in HMC-1 cells and in neoplastic MCs from MCL patients. In humans, Aurora kinase A and Plk1 cooperate in different spatiotemporal contexts regulating mitosis and cytokinesis, and their kinase activity is reciprocally regulated [29]. Hence, we reasoned that Plk1 inhibition might phenocopy the effects of Aurora kinase A inhibition. Indeed, a WB approach showed that Plk1 expression and activation parallel that of Aurora kinase A and inversely correlate with SETD2 protein levels. Moreover, the Plk1 inhibitor volasertib showed comparable efficacy to the Aurora kinase A inhibitor alisertib in HMC-1 cells and SETD2-deficient neoplastic MCs. Using real time RT-PCR and immunohistochemistry, Peter et al. [36] had already shown that Plk1 can be detected in an activated form in neoplastic MCs, and that either Plk1 silencing or pharmacological inhibition resulted in growth arrest and apoptosis in HMC-1 cells as well as in primary neoplastic MCs from SM patients. Interestingly, the LD_50_ values of the Plk1 inhibitor used by Peter et al. (BI-2536, whose chemical modification, aimed to improve the pharmacokinetic profile, later led to volasertib [37]) were identically low in HMC-1.1 and HMC-1.2, but varied greatly (from as low as 10 nM up to 1µM) in patients’ cells [36]. This different sensitivity might have reflected different SETD2 levels. In a subsequent study, Peter et al. [38] found that Aurora kinase A is expressed at the mRNA and protein level in neoplastic MCs (although its activity was not assessed) and that Aurora kinase A knock-down decreased proliferation in HMC-1.2 cells. They also showed that R763 (an inhibitor of Aurora kinases A and B, KIT, BTK, AKT and STAT5) induced growth inhibition and apoptosis in neoplastic MCs, either as single agent or in combination with midostaurin or dasatinib. Again, the LD_50_ values in primary patient cells were highly variable (from less than 1 nM to more than 1 µM). Our hypothesis that the inhibition of Aurora kinase A/Plk1 axis exerts its efficacy via SETD2/H3K36Me3 rescue would support and provide an explanation to Peter et al.’s findings [36, 38].

## Conclusions

The present study sheds further light on a novel mechanism of SETD2 loss of function. Our findings concur to depict a model whereby the aberrant expression and activation of Aurora kinase A triggers MDM2-mediated ubiquitination of SETD2, leading to enhanced proteasomal degradation and consequently to H3K36Me3 deficiency. Moreover, our study highlights promising therapeutic strategies for AdvSM patients based on easily repurposable targeted agents. At least in vitro, proteasome inhibitors, Aurora kinase A inhibitors and Plk1 inhibitors were found to be at least as effective as avapritinib, and the combinations showed significant advantages at very low doses of drugs. We thus propose that these inhibitors be further explored as a salvage option in patients with non-genomic SETD2 loss of function who fail or do not tolerate midostaurin or avapritinib.

## Supplementary Information

**Additional file 1: Supplementary methods.**

## Data Availability

Data and materials described in this manuscript, including all relevant raw data, will be freely available upon request to any researcher wishing to use them for non-commercial purposes.

## Abbreviations

SM: Systemic mastocytosis
MCs: Mast cells
BM: Bone marrow
AdvSM: Advanced SM
SM-AHN: SM with an associated hematologic neoplasm
ASM: Aggressive SM
MCL: MC leukemia
ISM: Indolent SM
SSM: Smoldering SM
WHO: World health organization
H3K36Me3: Trimethylation of histone H3 at lysine 36
WB: Western blotting
co-IP: Co-immunoprecipitation
PI: Propidium iodide
RNAi: RNA interference
LD_50_: Lethal dose 50
ccRCC: Clear cell renal cell carcinoma
SiRNA: Short interfering RNA
Plk1: Polo like kinase 1
NC: Negative control
