# Supplementary material for: SETD2 non genomic loss of function in advanced systemic mastocytosis is mediated by an Aurora kinase A/MDM2 axis and can be therapeutically targeted
Source: Biomark Res. 2023 Mar 10;11:29. doi: 10.1186/s40364-023-00468-7 (PMC9999558; doi:10.1186/s40364-023-00468-7)
Supplement: Supplementary file 1 — Additional file 1: Supplementary methods. [file 40364_2023_468_MOESM1_ESM.docx]

**Supplementary methods**

**Cell lines and patients samples.**

The HMC-1 cell line was generated from a patient with MCL,^1,2^ and has two subclones: HMC-1.1 harboring the V560G *KIT* mutation, and HMC-1.2 harboring both the V560G and the D816V *KIT* mutations.^3^ As we have previously reported, both subclones lack SETD2 protein and H3K36Me3 as a result of proteasome-mediated degradation.^4^ The ROSA^KIT D816V^ cell line was obtained by transfecting CD34^+^ umbilical cord blood cells with a lentiviral vector expressing the *KIT* D816V gene and was cultured as reported by Saleh et al.^19^ ROSA^KIT D816V^ cells express detectable levels of SETD2 protein and H3K36Me3.

For Western blotting (WB), bone marrow mononuclear cells from patients with ISM (n=11) and AdvSM (n=14) were used. Patients were diagnosed and classified according to WHO criteria.^5-7^ For drug treatment experiments, primary neoplastic MCs isolated from 3 patients diagnosed with MCL were used.

**Drugs**

Bortezomib (cat. S1013), carfilzomib (cat. S2853), ixazomib (cat. S2180) were used to inhibit proteasome activity; SP141(cat. S0901) was used to selectively inhibit MDM2; alisertib (cat. S1133) was used to selectively inhibit Aurora kinase A; volasertib (cat. 2235) was used to inhibit Plk-1 and avapritinib (cat. 8553) was used to inhibit the KIT mutation, D816V. All drug from Selleckchem were solubilized in DMSO at a stock concentration of: 1 mM for bortezomib, carfilzomib and ixazomib and 10 mM for other inhibitors.

**RNA interference (RNAi).**

Cells were plated at a density of 10^5^ cells/mL in a 6-well plate. 24 hours later, cells were transfected using 100 pM siRNA positive control duplex, or 100 pM negative control duplex or 100 pM of the specific siRNAs (Integrated DNA Technologies), mixed in 500 μL of serum-free medium and 7.5 μL of TransIT-siQUEST Reagent (Mirus). The siRNA duplex sequences selected (among three different sequences tested) to silence S*ETD2*, *MDM2* and *AURKA* were 5’-AAGAAUAAAUCUCAUCGAGAUAUTA-3’, 5’-CUUUACAUGUGCAAAGAAGCUAAAG.3’, and 5’-GACAGGAACAUGCUACUGAAGUUTA-3’, respectively. Twenty-four hours post-transfection, RNA was isolated using AllPrepDNA/RNA/Protein mini kit (Qiagen), and target transcript levels were measured by real time reverse transcriptase-quantitative polymerase chain reaction (RT-qPCR) on an ABI PRISM 7900HT instrument using the following TaqMan gene expression assays (ThermoFisher Scientific), *SETD2*: Hs01014784_m1; *MDM2*: Hs01066930_m1; *AURKA*: Hs01582072_m1. Relative expression was normalized to *GUSB* mRNA (Hs00939627_m1) and the negative control was used as baseline (100%). Twenty-four, 48 and 72 hours post-transfection, protein expression was assessed by WB.

**Co-immunoprecipitation/immunoblotting and Western blot analyses.**

WB was performed using 40 μg of whole cell lysates, separated on 10% Tris-glycine gels, transferred, and blotted. Immunoprecipitation (IP) was performed using 250 µg of whole cell lysates in the presence of Cyanogen bromide (CNBr)-activated sepharose 4B (GE Healthcare) conjugated with an anti-SETD2 antibody (Abnova). IP products were resolved by SDS-PAGE, blotted and labeled with primary and secondary antibodies.

**Clonogenic assay**

In HMC-1 cells we evaluated the reduction of colony (generated in 0.9% methylcellulose supplemented with 30% fetal calf serum) number in the presence of increasing doses of bortezomib (0.25-1 nM), carfilzomib (0.1-0.5 nM), ixazomib (0.5-2 nM), SP141 (0.1-0.3 µM), alisertib (25-100 nM), volasertib (10-50 nM) and avapritinib (5-25 nM) alone or in combination with alisertib (25-100 nM). In ROSA^KIT D816V^ cells we evaluated the reduction of colonies in the presence of increasing doses of bortezomib (0.2-1 nM), carfilzomib (0.1-0.4 nM), ixazomib (0.2-1 nM) and avapritinib (5-25 nM), alone or in combination with alisertib (25-100 nM). In primary cells from AdvSM patients we evaluated colony reduction in the presence of increasing doses of bortezomib (0.2-1 nM), carfilzomib (0.1-0.4 nM), ixazomib (0.2-1 nM), alisertib (50-250 nM) and volasertib (50-300 nM). After 10 days of incubation at 37°C in fully humidified atmosphere and 5% CO_2_, colonies were counted, and nonlinear regression analyses were used to calculate the lethal dose (LD_50_) of the different drugs.

**Supplementary References**

1. Butterfield JH, Weiler D, Dewald G, Gleich GJ. Establishment of an immature mast cell line from a patient with mast cell leukemia. *Leuk Res*. 1988;12:345.
2. Saleh R, Wedeh G, Herrmann H, et al. A new human mast cell line expressing a functional IgE receptor converts to tumorigenic growth by KIT D816V transfection. *Blood*. 2014;124:111.
3. Sundstrom M, Vliagoftis H, Karlberg P, et al. Functional and phenotypic studies of two variants of a human mast cell line with a distinct set of mutations in the c-kit proto-oncogene. *Immunology*. 2003;108:89.
4. Martinelli G, Mancini M, De Benedittis C, et al. SETD2 and histone H3 lysine 36 methylation deficiency in advanced systemic mastocytosis. *Leukemia*. 2018;32:139.
5. Valent P, Horny HP, Escribano L, et al. Diagnostic criteria and classification of mastocytosis: a consensus proposal. *Leuk Res*. 2001;25:603.
6. Valent P, Akin C, Metcalfe DD. Mastocytosis: 2016 updated WHO classification and novel emerging treatment concepts. *Blood*. 2017;129:1420.
7. Valent P, Akin C, Arock M, et al. Definitions, criteria and global classification of mast cell disorders with special reference to mast cell activation syndromes: a consensus proposal. *Int Arch Allergy Immunol*. 2012;157:215.
